# Association between Depressive Mood, Antidepressant Therapy and Neuropsychological Performances: Results from a Cross-Sectional Study on Elderly Patients

**DOI:** 10.3390/brainsci14010054

**Published:** 2024-01-06

**Authors:** Maristella Belfiori, Francesco Salis, Giorgia Demelas, Antonella Mandas

**Affiliations:** 1 Department of Medical Sciences, and Public Health, University of Cagliari, SS 554 Bivio Sestu, Monserrato, 09042 Cagliari, Italy; francesco-salis@tiscali.it (F.S.); giorgia.dem@gmail.com (G.D.); amandas@unica.it (A.M.); 2 Department of Biomedical Sciences, University of Cagliari, 09042 Cagliari, Italy; 3 University Hospital “Azienda Ospedaliero-Universitaria” of Cagliari, 09127 Cagliari, Italy

**Keywords:** elderly, depressive mood, cognitive impairment, Repeatable Battery for the Assessment of Neuropsychological Status (RBANS), 15-item Geriatric Depression Scale (GDS-15)

## Abstract

Background: Currently, the global demographic landscape is undergoing a transformative shift towards an increasingly aging population. This leads to an increase in chronic pathologies, including depression and cognitive impairment. This study aimed to evaluate the association between depressive mood, whether in treatment or not, and cognitive capacities, assessed using the Repeatable Battery for the Assessment of Neuropsychological Status (RBANS). Methods: This study included 259 subjects, aged 65 years or older, evaluated at the Geriatric Outpatient Service of the University Hospital of Monserrato, Cagliari, between July 2018 and May 2022, who experienced subjective depressive mood and/or cognitive deficits. Results: Only 25.1% of the sample showed no cognitive impairment on the RBANS. Education was a significant regressor of the RBANS Total Scale scores (*p* < 0.0001) and was negatively associated with mood deflection (r = −0.15, *p* = 0.0161). Subjects with depressive mood had more impaired attention and visuospatial/constructional abilities compared to untreated euthymic patients. Post-hoc analysis, conducted with the Conover test, showed that untreated euthymic patients (GDS-15 ≤ 5, group 2) had a higher score on the RBANS total scale than patients with mood deflection (GDS-15 > 5, group 1), and treated euthymic patients (GDS-15 ≤ 5, group 3). Finally, different logistic regression analyses revealed a significant negative coefficient for GDS as a regressor of the RBANS total scale (coefficient: −0.04, *p* = 0.0089), visuospatial/constructional abilities (coefficient: −0.03, *p* = 0.0009), language (coefficient: −0.05, *p* = 0.0140), and attention (coefficient: −0.05, *p* < 0.0001). Conclusions: Our analysis demonstrated that “naturally” euthymic people show better cognitive performances than people with depressive mood and subjects with acceptable mood due to antidepressants. Furthermore, the gender-based difference observed in the language domain suggests the potential utility of incorporating an alternative category for male patients in the Semantic Fluency test.

## 1. Background

Currently, the global demographic landscape is undergoing a transformative shift towards an increasingly aging population. It is estimated that in Europe, between 2015 and 2050, the population over 60 years of age will double, going from 12% to 22% [1], while the elderly over 80 will quadruple [2]. This demographic phenomenon is to be correlated to the confluence of economic and social changes, such as advancements in healthcare, improved living conditions, and declining birth rates [3]. Significant consequences are associated with the increasingly older population: advanced age has a higher likelihood of multiple health conditions co-occurring, with a rise in chronic conditions, which is associated with increased resources demanded.

Among these, depression and cognitive impairment have a high prevalence in the aged population. According to the literature [4], the prevalence of depression in older adults is significant, at approximately 35.1%. Also, in Europe depression in the geriatric population is one of the most common mental disorders [5,6] with the highest prevalence rates in France, Italy, and Spain [7]. However, late-life depression is still under-diagnosed, due to the exclusion of the elderly (over 85 years of age) from epidemiological studies [8], atypical symptoms, characterized by somatization, anxiety, suicidal ideation, apathy and emotional coercion [9,10] and social stigma, especially in the male population, who tend to be less inclined to ask for help [11,12].

Neurocognitive disorders are among the most common causes of morbidity and mortality in the elderly population, with a worldwide prevalence of dementia in 2010 amounting to 35.5 million, destined to double every 20 years [13,14]. According to the Diagnostic and Statistical Manual of Mental Disorders (DSM-5), we distinguish three clinical entities of neurocognitive disorder: delirium, major neurocognitive disorder, and mild neurocognitive disorder [15,16,17]. The last one, representing a “symptomatic pre-dementia stage”, is marked by objective impairment in cognition that is not severe enough to interfere with daily activities [18]. Neurocognitive disorders present a complex relationship with depression, such that the former can represent a risk factor for the latter and/or vice versa [19,20,21]. This association is stronger in older patients, where depression tends to have a later onset [22,23], and is characterized by long-lasting episodes [24]. Cognitive impairment is also a risk factor for the onset of depression; indeed, according to a recent review, depression among patients with mild cognitive impairment is higher than that among normal adults [25].

The impairment of cognitive function in patients suffering from depression is correlated not only with the dysfunction of the limbic-cortical network [26,27] and peripheral inflammation [28], but also with a reduction in the volume of some brain structures, such as the hippocampus, involved in the storage of memory information [9,29]. Antidepressant therapy, in addition to restoring the reduced levels of serotonin and/or noradrenaline (monoaminergic hypothesis) [30], can improve neuronal function through partial recovery of neuronal hypotrophy (neurotrophic hypothesis) [30], with consensual modulation not only of the affective but also of the cognitive sphere. However, the results in the literature are conflicting: the effect of antidepressant therapy on the cognitive function of patients with depression is controversial, as some studies suggest a greater risk of cognitive decline [31] and other improvements in cognitive functioning [32,33], although many studies are limited by a small number of patients or the use of non-sensitive cognitive assessment.

Nowadays, the neuropsychology of late-onset depression remains poorly understood. Although numerous studies describe cognitive functioning in these patients, few of them offer a comprehensive assessment of cognitive domains [33,34]. Following this line of research, this study aims to evaluate the association between depressive mood, whether in treatment or not, and neuropsychological performances.

## 2. Materials and Methods

### 2.1. Participants and Procedure Sections

This cross-sectional study was conducted on a convenience sample of 259 participants, aged 65 years or older, including 150 (57.9%) women.

We recruited patients who came to the Geriatric Outpatient Service of the University Hospital of Monserrato, Cagliari, from July 2018 to May 2022 for a suggestive medical history of depressive mood and/or cognitive deficits, reported by the patients themselves and/or their caregivers.

The enrolled patients were subjected by trained geriatricians to mood assessment with the Geriatric Depression Scale (GDS-15) and cognitive assessment with the Repeatable Battery for the Assessment of Neuropsychological Status (RBANS).

Inclusion criteria: age ≥ 65 years; being subjected to GDS-15 and RBANS.
Exclusion criteria: age < 65 years; informed consent not provided.

To detect any gender differences, we created two groups post-hoc (male and female groups). Some of the participants (69 subjects) were under antidepressant treatment, and, in order to see the association between mood and cognitive performance, we divided the sample into three groups post-hoc: untreated patients with depressive mood (group 1), untreated euthymic patients (group 2) and treated euthymic patients (group 3).

### 2.2. Assessment

The enrolled participants underwent the following evaluations:

▪ **Repeatable Battery for the Assessment of Neuropsychological Status (RBANS)**, a neuropsychological battery that evaluates five cognitive domains (immediate memory, visuospatial/constructional abilities, language, attention, and delayed memory), each with an index score (RBANS-IS), the sum of which is converted into the total scale index (RBANS-TIS). A RBANS-TIS between 85 and 70 indicates a probable cognitive deficit, while a score < 70 indicates cognitive impairment [35,36].
▪ **Geriatric Depression Scale (GDS-15)**, a mood screening tool composed of 15 questions, the answers to which are yes or no. The total score ranges from 0 (not depressive mood) to 15 (depressive mood). A score above 5 is indicative of a depressive mood [37,38,39].

### 2.3. Statistical Analysis

Variables were expressed as frequencies and percentages, or medians and interquartile ranges, in consideration of their asymmetrical distribution. Continuous variables were compared using the Mann–Whitney test. Categorical variables were compared using the chi-squared test (χ²). The Kruskal–Wallis test was used to compare patients with mood deflection (group 1), untreated euthymic patients (group 2), and treated euthymic patients (group 3). The Conover test was performed for post-hoc analysis. Multivariable analysis was performed with multiple regressions and logistic regression—stepwise method (variables whose coefficients presented *p*-values > 0.1 have been excluded from the model). The results were reported as *p*-values with reference to 95% confidence intervals (C.I.). MedCalc software (Version 19.5, Ostend, Belgium) was used for statistical analysis.

## 3. Results

The study included 259 subjects, aged 65 years or older (median age: 75 years, range: 70–78 years), of whom 150 (57.9%) were women. The participants’ characteristics are presented in Table 1. Genre differences are illustrated in Table 2.

In accordance with the GDS-15, 161 patients (62.20%) received a score equal to or less than 5 indicating normal mood, while the remaining 98 (37.80%) obtained a score above 5, indicating mood deflection.

**Figure 1:**
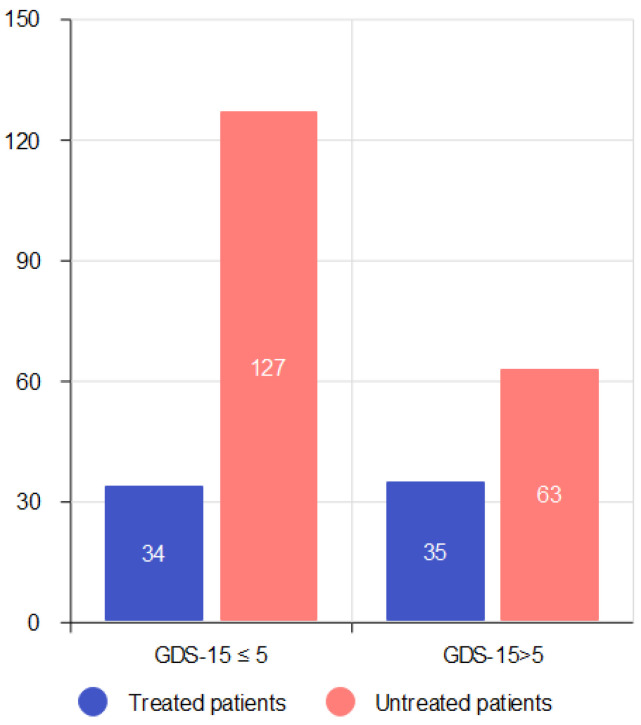
GDS-15 in treated and untreated patients GDS-15, Geriatric Depression Scale-15 items.

Of the enrolled patients, 69 subjects (26.6%) were receiving antidepressant therapy. To verify the association between the GDS-15 variable and antidepressant treatment, we performed a Chi-squared test, as shown in Figure 1. We observed that 34 (49.28%) patients under antidepressant treatment had normal scores on the GDS-15 (≤5), so they showed benefits in terms of fewer depressive symptoms (χ^2^: 6.615, *p* = 0.0101). The majority of patients receiving antidepressant therapy were taking selective serotonin reuptake inhibitors (SSRIs) (71%), followed by selective serotonin and norepinephrine reuptake inhibitors (SNRIs) (11.6%), tricyclic antidepressants (ADTs) (7.3%), specific noradrenergic and serotoninergic antidepressants (NaSSA) (4.3%), serotonin antagonist and reuptake inhibitors (SARI) (1.45%), serotonin modulators and stimulators (SMS) (1.45%), and combinations of antidepressants (2.9%).

In the assessment of cognitive function using the RBANS-TIS, only a small proportion of patients (25.1%) scored within the normal range (85+), while the majority (74.9%) exhibited poor cognitive scores (<85).

In order to explore education and gender influence on cognitive abilities, we performed a multiple regression, which revealed that education (*p* < 0.0001) was a significant regressor of the RBANS-TIS, while gender’s coefficient was nonsignificant (Table 3).

In addition, in order to consider whether education could also be associated with depressive mood, we performed a Pearson’s correlation, which revealed that education level was negatively associated with mood deflection (r = −0.15, *p* = 0.0161)

Following the aim of the study, RBANS-IS and RBANS-TIS scores were compared in people with deflected and normal moods using the Mann–Whitney test. This comparison is presented in Table 4, which shows that only attention and visuospatial/constructional abilities are more impaired in subjects with depressive mood.

**Figure 2:**
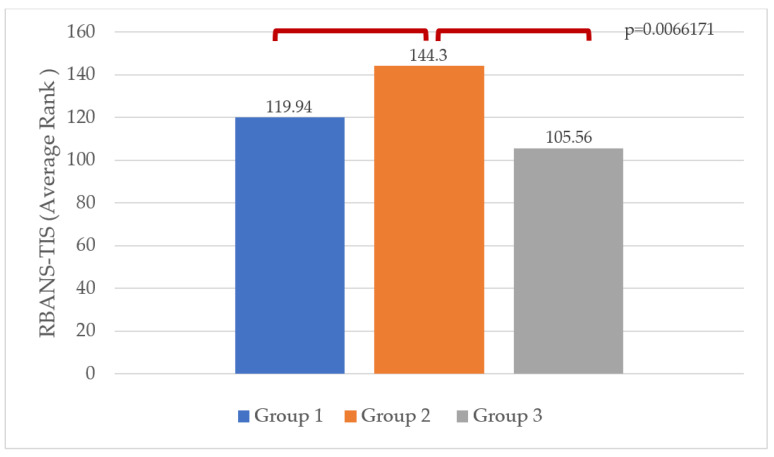
Kruskal–Wallis and Conover tests. RBANS-TIS, Repeatable Battery for the Assessment of Neuropsychological Status Total Score; group 1: untreated patients with depressive mood; group 2: untreated euthymic patients; group 3: treated euthymic patients.

To explore the impact of antidepressant therapy on cognitive abilities, we divided the population into three distinct groups: group 1 consisted of individuals experiencing a depressive mood (GDS > 5) but not currently receiving any form of treatment; group 2 consisted of untreated individuals with a normal mood (GDS ≤ 5); group 3 consisted of individuals with a normal mood (GDS ≤ 5) who were receiving effective antidepressant treatment. Using the Kruskal–Wallis test, we examined the differences in the RBANS-TIS according to mood deflection (as presented in Figure 2. Significant differences (*p* = 0.0066171) were found among the three groups. Post-hoc analysis, conducted with the Conover test, showed that the second group had a higher score on the RBANS-TIS than the first and third groups (Figure 2).

Finally, different logistic regression analyses were conducted to determine if mood (GDS-15 as an independent variable, divided according to the 5-point threshold) had an influence on RBANS-TIS, and each RBANS-IS (dependent variables). The analyses revealed a significant negative coefficient for GDS as a regressor of RBANS TIS (coefficient: −0.04, *p* = 0.0089), visuospatial/constructional abilities (coefficient: −0.03, *p* = 0.0009), language (coefficient: −0.05, *p* = 0.0140), and attention (coefficient: −0.05, *p* < 0.0001). The other regressions gave nonsignificant coefficients and were not displayed.

## 4. Discussion

It is documented by several studies that cognitive impairment and depression are associated in adult and elderly subjects [24,40]. However, in elderly patients, the association between these two problems is still complex and incomplete [21,41,42,43]. Therefore, our study aimed to evaluate the association between depressive mood and cognitive impairment in a population of 259 subjects aged 65 years or older.

In our study, only 25.1% of the patients showed intact cognitive function, and 62.20% had adequate mood.

We found that age and gender had no impact on GDS-15 scores, which aligns with the existing literature [44]. However, we observed a correlation between education level and deflected mood (*p* = 0.0159). Our results indicated that individuals with higher education levels achieved higher scores on the GDS-15, even if the correlation we found was weak, consistent with previous research [19], and on RBANS-TIS. In light of our study, attending school has a noteworthy influence on both mood and cognitive performance. Specifically, education has been found to promote more efficient cognitive processing and increase cognitive reserve [45]. Additionally, it is important to mention that education is correlated with greater access to screening and treatment for depression [46].

Also, although no significant differences in RBANS-TIS were observed in males and females, visuospatial/constructional skills showed better performances in men (*p* = 0.0012), and language domain (*p* = 0.0015) in women. Indeed, according to the literature, there is genre variability among regional cortical volumes [47,48], which may account for differences in cognitive abilities between males and females. The larger sizes of primary visual and visuospatial association areas in males might explain their superior performance in visuospatial tasks. Conversely, the relative simplicity of female participants in categorizing elements such as fruits and vegetables in the Semantic Fluency test, as previously demonstrated in research, may be linked to increased grey matter volume in the auditory and language-related regions of the left hemisphere, which are more developed in females [47,48].

To investigate differences in cognitive performances between patients with or without depressive mood, regardless of the therapy, the population was divided into three groups (depressed patients, euthymic patients because of the treatment, and patients with normal mood not on treatment). Patients without depressive mood and treatment revealed overall better cognitive performances (*p* = 0.006617). To the best of our knowledge, this aspect represents an original element in geriatric literature since it shows that there is no significant difference in cognitive performance between people with depressive mood and euthymic people taking antidepressants. Moreover, for equal GDS-15 scores, people who achieve that score “naturally” (without the help of antidepressants) have better cognitive performances.

To investigate more precisely which cognitive domains were most influenced by depressive mood, we found that the second was independently associated with worse visuospatial/constructional abilities (*p* = 0.0009), language (*p* = 0.0140), and attention (*p* < 0.0001).

## 5. Conclusions

In conclusion, our study found that the RBANS is reliable in identifying geriatric patients with cognitive impairment.

Despite no significant difference in cognitive performance considering depressive or not-depressive mood, we found that the difference emerged when considering depression treatment. In particular, “naturally” euthymic people show better cognitive performances than people with depressive mood and subjects with acceptable mood due to antidepressants. Finally, males achieving worse performances in language can suggest the future inclusion of alternative semantic categories for male patients.

However, we must acknowledge the methodological limitations of this study. As this was a monocentric cross-sectional study, there may be limitations to the generalizability of our findings, which calls for the need to adopt a longitudinal design in future research.

## Figures and Tables

**Table 1:** Characteristics of enrolled patients.

	Patients (N = 259)	
	Median (IQR)	Min–Max
*Age (Years)*	75 (70–78)	65–87
*Education level (Years)*	5.5 (5–13)	0–23
*GDS-15*	4 (2–7)	0–15
*RBANS TIS*	75 (68–84.5)	46–131
*Immediate memory RBANS-IS*	71 (54–90)	40–128
*Delayed Memory RBANS-IS*	87 (80.5–93.5)	43–146
*Language RBANS-IS*	80 (73.5–82)	40–110
*Attention RBANS-IS*	74 (62–89)	40–137
*Visuospatial/* *Constructional RBANS-IS*	95 (83–109)	40–140

IQR, interquartile range; GDS-15, Geriatric Depression Scale-15 items; RBANS, Repeatable Battery for the Assessment of Neuropsychological Status; TIS: total score; IS: index.

**Table 2:** Characteristics of enrolled patients and gender differences.

	Gender M (N. 109-42.1%)	Gender F (N. 150-57.9%)	
	Median (IQR)	Median (IQR)	Mann–Whitney Test *p*
Age (Years)	75 (70–79)	74 (71–78)	0.3667
Education level (Years)	8 (5–13)	5 (5–13)	**0.0232**
GDS-15	4 (2–6)	5 (3–8)	**0.0385**
RBANS TIS	75 (68–86)	75 (68–81)	0.3797
Immediate memory RBANS-IS	73 (55–87)	71 (52–91)	0.9143
Delayed Memory RBANS-IS	85 (82–92)	87 (80–95)	0.6896
Language RBANS-IS	80 (72–82)	80 (74–82)	**0.0310**
Attention RBANS-IS	77 (65–89)	74 (58–88)	0.2244
Visuospatial/ Constructional RBANS-IS	100 (85–114)	94 (82–103)	**0.0177**

IQR, interquartile range; M, male gender; F, female gender; GDS-15, Geriatric Depression Scale-15 items; RBANS, Repeatable Battery for the Assessment of Neuropsychological Status; TIS: total score; IS: index.

**Table 3:** Multiple regression—stepwise method (y = RBANS-TIS).

	RBANS-TIS		
Variables	Coefficient	*t*	*p*
Education level (Years)	1.60	0.56	**<0.0001**
Gender	0.92	9.14	0.5784

RBANS, Repeatable Battery for the Assessment of Neuropsychological Status; TIS: Total Scor

**Table 4:** Comparison between GDS-15 and the five cognitive domains of RBANS and Total Scale.

	GDS-15 ≤ 5 (N. 161)	GDS-15 > 5 (N. 98)	Mann–Whitney
	Median (IQR)	Median (IQR)	
RBANS TIS	75 (69–86)	74.5 (66–81)	*p* = 0.0915
Immediate memory RBANS-IS	72 (53.75–90,25)	71 (54–90)	*p* = 0.9257
Delayed Memory RBANS-IS	87 (81–94)	85 (80–93)	*p* = 0.6724
Language RBANS-IS	80 (75–82)	80 (70–82)	*p* = 0.1184
Attention RBANS-IS	77 (64.75–91)	69 (54–81)	***p* = 0.0009**
Visuospatial/ Constructional RBANS-IS	96 (86–111)	93.5 (76–106)	***p* = 0.0415**

IQR, interquartile range; RBANS, Repeatable Battery for the Assessment of Neuropsychological Status; TIS: total score; IS: index; GDS: Geriatric Depression Scale.

## Data Availability

The raw data used and/or analyzed during this current study will be made available upon reasonable request. The data are not publicly available due to privacy restrictions.

## References

[1] Besora-Moreno M., Llauradó E., Tarro L., Solà R. (2020). Social and Economic Factors and Malnutrition or the Risk of Malnutrition in the Elderly: A Systematic Review and Meta-Analysis of Observational Studies. Nutrients.

[2] Katsas K., Mamalaki E., Kontogianni M.D., Anastasiou C.A., Kosmidis M.H., Varlamis I., Hadjigeorgiou G.M., Dardiotis E., Sakka P., Scarmeas N. (2020). Malnutrition in older adults: Correlations with social, diet-related, and neuropsychological factors. Nutrition.

[3] Sadovnichy V., Akaev A., Ilyin I., Malkov S., Grinin L., Korotayev A. (2023). Reconsidering the Limits to Growth. World-Systems Evolution and Global Futures.

[4] Hu T., Zhao X., Wu M., Li Z., Luo L., Yang C., Yang F. (2022). Prevalence of depression in older adults: A systematic review and meta-analysis. Psychiatry Res..

[5] Copeland J.R., Beekman A.T., Braam A.W., Dewey M.E., Delespaul P., Fuhrer R., Hooijer C., Lawlor B.A., Kivela S.L., Lobo A. (2004). Depression among older people in Europe: The EURODEP studies. World Psychiatry.

[6] Solhaug H.I., Romuld E.B., Romild U., Stordal E. (2012). Increased prevalence of depression in cohorts of the elderly: An 11-year follow-up in the general population—The HUNT study. Int. Psychogeriatr..

[7] Castro-Costa E., Dewey M., Stewart R., Banerjee S., Huppert F., Mendonca-Lima C., Bula C., Reisches F., Wancata J., Ritchie K. (2007). Prevalence of depressive symptoms and syndromes in later life in ten European countries: The SHARE study. Br. J. Psychiatry.

[8] Beekman A.T., Geerlings S.W., Deeg D.J., Smit J.H., Schoevers R.S., de Beurs E., Braam A.W., Penninx B.W., van Tilburg W. (2002). The natural history of late-life depression: A 6-year prospective study in the community. Arch. Gen. Psychiatry.

[9] Alexopoulos G.S. (2005). Depression in the elderly. Lancet.

[10] Altamura A.C., Bassetti R., Santini A., Frisoni G.B., Mundo E. (2004). Emotional withdrawal, CT abnormalities and drug response in late life depression. Prog. Neuropsychopharmacol. Biol. Psychiatry.

[11] Luppa M., Sikorski C., Luck T., Ehreke L., Konnopka A., Wiese B., Weyerer S., König H.H., Riedel-Heller S.G. (2012). Age- and gender-specific prevalence of depression in latest-life—Systematic review and meta-analysis. J. Affect. Disord..

[12] Blazer D.G., Steffens D.C., Busse E.W. (2007). Essential of Geriatric Psychiatry.

[13] McDonald W.M. (2017). Overview of Neurocognitive Disorders. Focus.

[14] Prince M., Bryce R., Albanese E., Wimo A., Ribeiro W., Ferri C.P. (2013). The global prevalence of dementia: A systematic review and metaanalysis. Alzheimers Dement..

[15] Sachdev P.S., Blacker D., Blazer D.G., Ganguli M., Jeste D.V., Paulsen J.S., Petersen R.C. (2014). Classifying neurocognitive disorders: The DSM-5 approach. Nat. Rev. Neurol..

[16] Wilson J.E., Mart M.F., Cunningham C., Shehabi Y., Girard T.D., MacLullich A.M.J., Slooter A.J.C., Ely E.W. (2020). Delirium. Nat. Rev. Dis. Primers.

[17] Bo M., Porrino P., Di Santo S.G., Mazzone A., Cherubini A., Mossello E., Bianchetti A., Musicco M., Ferrari A., Ferrara N. (2019). Italian Study Group on Delirium (ISGoD). The association of indwelling urinary catheter with delirium in hospitalized patients and nursing home residents: An explorative analysis from the “Delirium Day 2015”. Aging Clin. Exp. Res..

[18] Yao S., Liu Y., Zheng X., Zhang Y., Cui S., Tang C., Lu L., Xu N. (2020). Do nonpharmacological interventions prevent cognitive decline? a systematic review and meta-analysis. Transl. Psychiatry.

[19] Palmer K., Di Iulio F., Varsi A.E., Gianni W., Sancesario G., Caltagirone C., Spalletta G. (2010). Neuropsychiatric predictors of progression from amnestic-mild cognitive impairment to Alzheimer’s disease: The role of depression and apathy. J. Alzheimer’s Dis..

[20] Chan W.C., Lam L.C., Tam C.W., Lui V.W., Leung G.T., Lee A.T., Chan S.S., Fung A.W., Chiu H.F., Chan W.M. (2011). Neuropsychiatric symptoms are associated with increased risks of progression to dementia: A 2-year prospective study of 321 Chinese older persons with mild cognitive impairment. Age Ageing.

[21] Siafarikas N., Selbaek G., Fladby T., Šaltytė Benth J., Auning E., Aarsland D. (2018). Frequency and subgroups of neuropsychiatric symptoms in mild cognitive impairment and different stages of dementia in Alzheimer’s disease. Int. Psychogeriatr..

[22] Diniz B.S., Butters M.A., Albert S.M., Dew M.A., Reynolds C.F. (2013). Late-life depression and risk of vascular dementia and Alzheimer’s disease: Systematic review and meta-analysis of community-based cohort studies. Br. J. Psychiatry.

[23] Barnes D.E., Yaffe K., Byers A.L., McCormick M., Schaefer C., Whitmer R.A. (2012). Midlife vs late-life depressive symptoms and risk of dementia: Differential effects for Alzheimer disease and vascular dementia. Arch. Gen. Psychiatry.

[24] Papakostas G.I. (2014). Cognitive symptoms in patients with major depressive disorder and their implications for clinical practice. J. Clin. Psychiatry.

[25] Ma L. (2020). Depression, Anxiety, and Apathy in Mild Cognitive Impairment: Current Perspectives. Front. Aging Neurosci..

[26] Raymond W.L. (2018). Depression.

[27] Drevets W.C. (2007). Orbitofrontal cortex function and structure in depression. Ann. N. Y Acad. Sci..

[28] Bortolato B., Carvalho A.F., Soczynska J.K., Perini G.I., McIntyre R.S. (2015). The Involvement of TNF-α in Cognitive Dysfunction Associated with Major Depressive Disorder: An Opportunity for Domain Specific Treatments. Curr. Neuropharmacol..

[29] Lyness J.M., King D.A., Conwell Y., Cox C., Caine E.D. (2000). Cerebrovascular risk factors and 1-year depression outcome in older primary care patients. Am. J. Psychiatry.

[30] Leng Y., Diem S.J., Stone K.L., Yaffe K. (2018). Antidepressant Use and Cognitive Outcomes in Very Old Women. J. Gerontol. A Biol. Sci. Med. Sci..

[31] Moraros J., Nwankwo C., Patten S.B., Mousseau D.D. (2017). The association of antidepressant drug usage with cognitive impairment or dementia, including Alzheimer disease: A systematic review and meta-analysis. Depress. Anxiety.

[32] Rosenblat J.D., Kakar R., McIntyre R.S. (2015). The Cognitive Effects of Antidepressants in Major Depressive Disorder: A Systematic Review and Meta-Analysis of Randomized Clinical Trials. Int. J. Neuropsychopharmacol..

[33] Butters M.A., Becker J.T., Nebes R.D., Zmuda M.D., Mulsant B.H., Pollock B.G., Reynolds C.F. (2000). Changes in cognitive functioning following treatment of late-life depression. Am. J. Psychiatry.

[34] Baune B.T., Suslow T., Engelien A., Arolt V., Berger K. (2006). The association between depressive mood and cognitive performance in an elderly general population—The MEMO Study. Dement. Geriatr. Cogn. Disord..

[35] Salis F., Costaggiu D., Mandas A. (2023). Mini-Mental State Examination: Optimal Cut-Off Levels for Mild and Severe Cognitive Impairment. Geriatrics.

[36] Randolph C., Tierney M.C., Mohr E., Chase T.N. (1998). The Repeatable Battery for the Assessment of Neuropsychological Status (RBANS): Preliminary clinical validity. J. Clin. Exp. Neuropsychol..

[37] Ponteri M., Pioli R., Padovani A., Tunesi S., De Girolamo G., Giunti O.S. (2007). RBANS: Repeatable Battery for the Assessment of Neuropsychological Status (Italian Adaptation).

[38] Yesavage J.A., Brink T.L., Rose T.L., Lum O., Huang V., Adey M., Leirer V.O. (1982). Development and validation of a geriatric depression screening scale: A preliminary report. J. Psychiatr. Res..

[39] Chiesi F., Primi C., Pigliautile M., Baroni M., Ercolani S., Boccardi V., Ruggiero C., Mecocci P. (2018). Is the 15-item Geriatric Depression Scale a Fair Screening Tool? A Differential Item Functioning Analysis Across Gender and Age. Psychol. Rep..

[40] Rock P.L., Roiser J.P., Riedel W.J., Blackwell A.D. (2014). Cognitive impairment in depression: A systematic review and meta-analysis. Psychol. Med..

[41] van den Kommer T.N., Dik M.G., Comijs H.C., Jonker C., Deeg D.J. (2012). Role of lipoproteins and inflammation in cognitive decline: Do they interact?. Neurobiol. Aging.

[42] Zandi T. (2004). Relationship between subjective memory complaints, objective memory performance, and depression among older adults. Am. J. Alzheimers Dis. Other Demen.

[43] Hook J.N., Han D.Y., Smith C.A. (2020). Repeatable Battery for the Assessment of Neuropsychological status (RBANS) and depressive complaints in older adults. Clin. Gerontol..

[44] Chiesi F., Primi C., Pigliautile M., Baroni M., Ercolani S., Paolacci L., Boccardi V., Mecocci P. (2018). Does the 15-item Geriatric Depression Scale function differently in old people with different levels of cognitive functioning?. J. Affect. Disord..

[45] Santos N.C., Costa P.S., Cunha P., Portugal-Nunes C., Amorim L., Cotter J., Cerqueira J.J., Palha J.A., Sousa N. (2014). Clinical, physical and lifestyle variables and relationship with cognition and mood in aging: A cross-sectional analysis of distinct educational groups. Front. Aging Neurosci..

[46] Taple B.J., Chapman R., Schalet B.D., Brower R., Griffith J.W. (2022). The Impact of Education on Depression Assessment: Differential Item Functioning Analysis. Assessment.

[47] Brun C.C., Leporé N., Luders E., Chou Y.Y., Madsen S.K., Toga A.W., Thompson P.M. (2009). Sex differences in brain structure in auditory and cingulate regions. Neuroreport.

[48] Kheloui S., Jacmin-Park S., Larocque O., Kerr P., Rossi M., Cartier L., Juster R.P. (2023). Sex/gender differences in cognitive abilities. Neurosci. Biobehav. Rev..

